# Supplementary material for: Predicting Rheumatoid Arthritis Development Using Hand Ultrasound and Machine Learning—A Two-Year Follow-Up Cohort Study
Source: Diagnostics (Basel). 2024 Jun 4;14(11):1181. doi: 10.3390/diagnostics14111181 (PMC11171890; doi:10.3390/diagnostics14111181)
Supplement: Supplementary file 1 [file diagnostics-14-01181-s001.zip › diagnostics-3026150-supplementary.pdf]

Supplementary information:

Abbreviations:

- **ACR:** American College of Rheumatology
- **ACPA:** Anti-cyclic citrullinated peptide antibody
- **CMC:** First carpometacarpal joint
- **CRP:** C-reactive protein
- **DARE:** Drug Abuse Resistance Education
- **DIP:** Distal interphalangeal joint
- **DMARD:** Disease-modifying antirheumatic drug
- **EULAR:** European League Against Rheumatism
- **ESR:** Erythrocyte sedimentation rate
- **GS:** Grey scale
- **IA:** Inflammatory arthritis
- **IP:** Interphalangeal joint of the thumb
- **KNN:** K nearest neighbor
- **MCP:** Metacarpophalangeal joint
- **ML:** Machine learning
- **MRI:** Magnetic resonance imaging
- **MTP:** Metatarsophalangeal joint
- **NN:** Neural network
- **OMERACT:** Outcome Measures in Rheumatoid Arthritis Clinical Trials
- **PD:** Power Doppler
- **PIP:** Proximal interphalangeal joint
- **PRF:** Pulse repetition frequency
- **RA:** Rheumatoid arthritis
- **RF:** Rheumatoid factor
- **ROC:** Receiver operating characteristic
- **SVM:** Support vector machine
- **US:** Ultrasound
- **WBC:** White blood cell count

Normal Values and Units:

- **WBC:** 10^9 cells/L
- **ESR:** Men ≤ 15 mm/hr, Women ≤ 20 mm/hr
- **RF:** IU/mL
- **CRP:** ≤ 0.80 mg/dL (standard laboratory methods)
- **Anti-CCP (ACPA):** > 18 IU/mL (considered positive)
- **Vitamin D:** nmol/L

Specific Analytical Methods:

- **IgM-RF:** Evaluated using turbidimetry with a calibrated HITACHI 912 autoanalyzer; a titer ≥ 20 IU/mL is considered positive.
- **ACPA:** Determined using an Awareness StatFax 4200 ELISA Microplate Reader; levels > 18 IU/mL are deemed positive per manufacturer guidelines.

Brief Explanations for Medical Terminologies:

- **Rheumatoid Arthritis (RA):** Rheumatoid arthritis is a chronic inflammatory disorder that primarily affects the joints, causing pain, swelling, stiffness, and loss of function. It can also affect other parts of the body, including the skin, eyes, lungs, and blood vessels. Early diagnosis and treatment are essential to prevent joint damage and disability.
- **Synovitis:** Synovitis refers to inflammation of the synovial membrane, which lines the joints. In rheumatoid arthritis, synovitis is a hallmark feature and involves the thickening of the synovial lining due to inflammation. It leads to joint pain, swelling, and stiffness.
- **Anti-Cyclic Citrullinated Peptide (anti-CCP) Antibodies:** Anti-CCP antibodies are autoantibodies produced by the immune system that target cyclic citrullinated peptides. Their presence in the blood is strongly associated with rheumatoid arthritis and can help diagnose the disease, especially in its early stages. Elevated levels of anti-CCP antibodies indicate increased disease severity and may predict more aggressive joint damage.

Definitions for Variables Mentioned in Tables 1 and 2:

- **WBC (White Blood Cell Count):** WBC is a measure of the total number of white blood cells in the blood. Elevated WBC counts may indicate inflammation or infection, which are common features of rheumatoid arthritis.
- **ESR (Erythrocyte Sedimentation Rate):** ESR is a blood test that measures the rate at which red blood cells settle at the bottom of a tube over a certain period. Elevated ESR levels suggest the presence of inflammation in the body, which is often seen in rheumatoid arthritis.
- **RF (Rheumatoid Factor):** RF is an autoantibody that targets the body's own tissues, particularly in rheumatoid arthritis. Elevated RF levels in the blood are associated with increased disease activity and severity.
- **Anti-CCP (Anti-Cyclic Citrullinated Peptide) Antibodies:** As explained earlier, anti-CCP antibodies are autoantibodies that target cyclic citrullinated peptides and are strongly associated with rheumatoid arthritis.

ACR/EULAR 2010 classification:

- The 2010 American College of Rheumatology/European League Against Rheumatism (ACR/EULAR) classification criteria are pivotal guidelines used in the diagnosis and classification of RA. Developed collaboratively by two leading rheumatology organizations, these criteria serve as a standardized framework for identifying individuals with RA based on clinical and laboratory features. They encompass a set of parameters, including clinical signs and symptoms, laboratory tests, and imaging findings, which are evaluated to determine the likelihood of RA diagnosis. By adhering to these criteria, clinicians can achieve consistency and accuracy in diagnosing RA, facilitating timely initiation of appropriate treatment strategies and improving patient outcomes[25].
